# Pediatric ACL Injuries: A Current Concepts Review

**DOI:** 10.3390/jcm15114253

**Published:** 2026-05-31

**Authors:** Caroline de Pesters, Tom Piscaer, Muhammad Deryl Ivansyah, Duncan Meuffels, Linda van den Berg, Franck Accadbled

**Affiliations:** 1 Department of Orthopaedics, Children’s Hospital, Toulouse University Hospital, 330 Avenue de Grande Bretagne, 31300 Toulouse, France; faccadbled@gmail.com; 2 Department of Orthopedics and Sports Medicine, Erasmus MC University Medical Center, Dr. Molewaterplein 40, 3015 GD Rotterdam, The Netherlands; t.piscaer@erasmusmc.nl (T.P.); d.meuffels@erasmusmc.nl (D.M.); l.e.m.vandenberg@erasmusmc.nl (L.v.d.B.); 3 Department of Orthopaedics and Traumatology, Dr. Cipto Mangunkusumo Hospital, Faculty of Medicine Universitas Indonesia, Jl. Salemba Raya No. 6, Central Jakarta 10430, Indonesia; deryl2006@gmail.com

**Keywords:** pediatric ACL, knee morphology, conservative management, ACL reconstruction, ACL repair, growth disturbances, graft remodeling, prevention, sports medicine

## Abstract

Background/Objectives: Anterior cruciate ligament (ACL) tears in children and adolescents are occurring at the intersection of skeletal growth and often early sports specialization, requiring a specialized approach, as pediatric ACL injury is not merely a scaled-down version of adult injury. Methods: This review synthesizes the current understanding of diagnostic protocols, evolution of knee morphology and neuromechanical risk factors characteristic of the pediatric population. It further examines the spectrum of specific management strategies including conservative approaches, primary repair and various reconstruction techniques, alongside rehabilitation, prevention and follow-up procedures. Results: In the diagnostic phase, pediatric-specific clinical and imaging findings must be carefully interpreted. Certain anatomical and neuromuscular characteristics seem to be linked to injury. Management remains complex, requiring a delicate balance between restoring stability and sparing bone growth. While conservative treatment may be attempted in specific cases, it must be promptly redirected toward surgical intervention if persistent instability occurs. Consensus on optimal surgical strategies remain impeded by the lack of robust evidence. Anterior cruciate ligament reconstruction (ACLR) still faces challenges such as growth disturbances, high graft failure, contralateral rupture rates and the biological process of graft remodeling. However, ACLR currently remains the gold standard compared to ACL repair. Tailored rehabilitation and robust prevention programs are needed. Conclusions: The management of ACL rupture in the pediatric population remains complex and constrained by important evidence gaps. Continued refinement of management strategies and future prospective, multicenter pediatric studies are needed.

## 1. Introduction

Anterior cruciate ligament (ACL) tears are increasingly documented in children and adolescents, where they occur in a specific context combining skeletal growth and often intensive and specialized sports practice from an early age [1,2]. Pediatric ACL injury is not merely a scaled-down version of adult injury. It is a distinct field that requires careful consideration. A summary of key clinical recommendations can be found in Appendix A.

## 2. Methods

### 2.1. Database Search and Selection

We conducted iterative PubMed (National Center for Biotechnology Information, Bethesda, MD, USA) searches between 1 June 2025, and 1 April 2026, guided by predefined research questions focusing on pediatric ACL injury topics. Search terms covered pediatric (children and adolescent) populations, risk factors, imaging, surgical or non-surgical management and outcomes and prevention. Additionally, reference lists of key studies and reviews were screened to identify influential studies not captured in the electronic search. Selection was based on relevance and methodological contribution to the predefined domains of the review, with emphasis on pediatric populations when available. As a non-systematic review, this work did not involve duplicate screening, formal risk-of-bias assessment or PRISMA reporting and may be subject to selection bias.

### 2.2. Illustration Design

Illustrations were designed by an independent illustrator using Procreate (Savage Interactive Pty Ltd., Hobart, TAS, Australia). The illustrations were developed under the supervision of the authors to ensure accuracy.

### 2.3. Terminology

Terminology was standardized as follows: skeletal maturity indicates closed physes, skeletal immaturity refers to open physes. Children are defined as <12 years old and adolescents as >12 years old, with the term pediatric patients encompassing both groups.

## 3. Epidemiology

In recent years, the incidence of ACL rupture in children and adolescents has shown a clear upward trend, as reported in several national registries [3,4,5]. This growing incidence is mainly attributed to increased exposure to competitive sports, early specialization and enhanced detection [5,6]. While rare in children under 12 years of age, the occurrence of ACL tears rises sharply throughout the teenage years with a peak in late adolescence. Boys are more frequently affected at younger ages [7], but girls surpass them with advancing age, particularly in pivoting sports [8]. A recent meta-analysis and systematic review reported an incidence rate per 1000 athlete-exposure of 0.084 in adolescent girls versus 0.060 in boys with a relative risk for girls-to-boys of 1.40 [9].This higher rate among teenage girls is likely driven by biomechanical, hormonal, and neuromuscular differences. Data regarding correlation between ACL rupture and ethnicity is limited [7]. Some studies have found an increased risk of ACL tear associated with higher BMI [10].

## 4. Initial Evaluation of an ACL Rupture

### 4.1. Medical History

The assessment of an ACL rupture is primarily based on the analysis of the initial symptoms. A detailed history of the trauma or event having led to the symptoms is relevant when possible. Most of these injuries occur in a sports setting [7]. Patients may present with acute pain, rapidly progressive knee joint swelling due to hemarthrosis and a feeling of knee instability. They may describe having felt a “pop” during initial trauma [11]. In young athletes with traumatic hemarthrosis of the knee, it is estimated that approximately 65% of cases correspond to an ACL rupture [12]. Joint locking may also be observed, particularly in the presence of an associated meniscal lesion.

### 4.2. Physical Examination

In acute settings, hemarthrosis is a common finding. To account for individual laxity in pediatric populations, clinical tests must be performed bilaterally. However, radiographic evaluation should first rule out any intra-articular fracture. The Lachman test is considered the gold standard for assessing anterior laxity. The anterior drawer and pivot-shift tests complete the assessment of instability [11,13]. Evaluating associated injuries is essential. This involves assessing the medial (MCL) and lateral (LCL) collateral ligaments as well as the integrity of the posterior cruciate ligament (PCL) and the postero-lateral corner (PLC). The evaluation of these associated structures relies heavily on tests established for the adult population. Various maneuvers can be used to uncover associated meniscal injuries [14]. The clinical assessment of lower-limb alignment [15] and leg-length discrepancies (LLDs) [16] serves to establish a fundamental baseline. This is crucial for long-term surveillance to detect any growth disturbances resulting from physeal injury.

### 4.3. Complementary Imaging

Anteroposterior and lateral radiographs of the knee should always be performed as a first step to identify any associated avulsion fracture. Magnetic resonance imaging (MRI) is the gold standard for confirming the radiological diagnosis of an ACL rupture and provides an assessment of associated lesions [17]. Preoperative evaluation of the lower limbs using long-leg radiographs or EOS (Electronic Optical Scan) imaging is considered essential, especially when surgical intervention is planned. This allows a precise and comprehensive assessment of baseline axial alignment and LLD.

### 4.4. Associated Lesions

#### 4.4.1. Meniscal Injuries

Meniscal injuries associated with primary ACL injury in pediatric populations are common and occur in 50–85% of patients [7,18,19,20]. Matava et al. [18] and Crippa et al. [19] found a correlation between an increased risk of meniscal injury and more advanced skeletal maturity and older age respectively. Conversely, Grassi et al. highlighted a higher prevalence of meniscal tear in patients with more than 1 year of remaining growth [20]. Lateral longitudinal tears seem to be more frequent in younger patients. As skeletal maturity approaches, the incidence of medial lesions rises, with ramp lesions being more frequently observed [20]. In adults, the posterior horn of the medial meniscus is preeminently affected [21]. This transition from lateral to medial injury patterns across age groups possibly reflects biomechanical differences.

#### 4.4.2. Other Associated Lesions

Concurrent injuries affect more than half of pediatric patients with ACL tears. This rate rises from 54–58% in early childhood to >70% among adolescents [22,23,24]. Up to 30% of ACL injuries involve chondral lesions [25]. Their prevalence and severity increases with delayed surgery, especially in younger patients [26]. MCL tears account for 2.3–3% of associated injuries [24,27] and are more common in contact trauma in adolescents [22]. LCL (0.11–0.5%) and PCL (0.4%) injuries are rarer and usually part of a complex injury pattern [24,27]. Although likely underdiagnosed, associated complete tears of at least one component of the PLC occur in 14% of cases [28]. While these lesions could lead to mediocre clinical outcomes by causing persistent rotatory instability, evidence within the pediatric population is currently lacking.

## 5. Evolution of Pediatric Knee Morphology

Pediatric ACL ruptures involve patients across a wide spectrum of ages and developmental stages. Consequently, it is essential to consider the distinct developmental patterns of both bony and soft tissues, as the knee undergoes substantial morphological changes.

### 5.1. Bone Growth

The distal femoral and proximal tibial physes play a major role in longitudinal growth. The knee is the joint contributing the most significantly to skeletal growth, with an average annual growth of approximately 2 cm. The distal femur accounts for about 60% of growth (~1.2 cm/year), while the proximal tibia contributes the remaining 40% (~0.8 cm/year) [29]. The knee growth plates show predictable ossification patterns on MRI that correlate with skeletal maturity [30].

### 5.2. Lower-Limb Alignment

It follows a predictable physiological progression from genu varum until approximately 18–24 months of age with a transition to maximum genu valgum by age 3–4. It then gradually reaches near-adult alignment, with a slight valgus of 1–3° by the age of 7 [15].

### 5.3. ACL Growth

The ACL morphology follows sex-specific and age-dependent changes throughout childhood [31,32]. ACL progressively and linearly lengthens until approximately 12 years in females and 14 years in males. This process occurs in three distinct phases and is independent of growth peaks. Growth averages a rapid 2.25 mm/year from ages 1.5 to 6, slows to a moderate 1.46 mm/year between ages 6 and 11.5, and begins to decelerate at age 11.75 before ceasing by age 18.5 [32]. ACL cross-sectional area (CSA) increases more pronouncedly until age 11 in girls and 12 in boys, before stabilizing from 11 to 14 years and 12 to 15 years, respectively. Following this phase, a slight decrease may occur [31,33]. Girls exhibit thicker ACLs between 0 and 6 years, whereas boys begin to demonstrate greater ACL thickness from 7 to 9 years, with statistically significant differences observed between 16 and 18 years [34]. Furthermore, ACL CSA-to-length ratio increases with age exclusively in boys [35]. Thus, despite similar ACL sizes between boys and girls at an early age, adolescent males have significantly longer and thicker ACLs compared to age-matched females [36]. ACL dimensions correlate with height and weight, whereas BMI shows only a weak association [34]. Notably, ACL area and intercondylar notch growth plateau earlier than longitudinal skeletal growth, suggesting they do not fully parallel skeletal maturation. In younger patients, the ACL is more oblique and anteriorly positioned. With advancing age, the ligament undergoes progressive verticalization in both sagittal and coronal planes. Concurrently, inclination of the intercondylar roof angle progressively diminishes [31].

### 5.4. Anatomical Development of the Immature Knee

The intercondylar notch width follows a characteristic growth pattern, increasing until 10–11 years of age, stabilizing until 13–14 years (earlier in girls than boys) and slightly decreasing thereafter. Notch width index (NWI) is highest in younger patients and progressively declines with age [37]. Anchustegui et al. evaluated the evolution of the medial (MTS) and lateral (LTS) tibial slope. Neither changed significantly with age [38]. Available data indicate that linear measurements of trochlear heights (lateral, medial and central) increase progressively with age. Trochlear morphological development appears to be complete around the age of 12 [39]. The resident’s ridge is a bony eminence serving as a surgical landmark as it defines the anterior border of the ACL’s femoral attachment. It is less delineated in younger patients and becomes more prominent with age [40].

## 6. Pediatric Risk Factors Associated with an ACL Injury

Studies have identified multiple morphological variations associated with ACL rupture in pediatric patients. However, findings present both consistencies and discrepancies. Notably, Micicoi et al. highlighted that more than 50% of healthy adult individuals exhibit at least one established morphological ACL risk factor [41]. This questions whether these parameters truly represent pathological anatomy. ACL injury risk in children and adolescents probably involves multifactorial anatomic variations rather than isolated morphological features. Furthermore, measurement methods and proposed thresholds vary across studies. Consequently, the following anatomical factors should be employed as complementary markers, useful for contextualizing risk rather than stand-alone screening tools.

### 6.1. Intercondylar Notch

Across the pediatric literature, a narrow intercondylar notch, typically defined by a decreased NWI and/or a reduced notch volume, appears to be associated with ACL injury [42]. In a 2025 systematic review, average NWI was 0.25 in the ACL-injured group versus 0.26 in the control group [42]. Similarly, Shin et al. showed that a higher NWI was protective ACL tears, with mean values of 23% in the injured group and 27% in controls [43]. Manhard et al. stated that pediatric patients with ACL tears show similar narrow NWI but that only adolescents demonstrated narrow notch height index, suggesting developmental differences [44].

### 6.2. Tibial Slope

In pediatric populations, current pooled evidence shows no statistically significant association between increased posterior tibial slope (overall PTS), MTS or LTS and primary ACL tear [45]. However, LTS is the only parameter that consistently trends toward significance [42,43,46]. Evidence is presently insufficient to establish a pediatric-validated LTS cutoff, with the only explicit, ROC-derived threshold being >4° [46]. Pradhan et al. provided crucial developmental context, demonstrating that LTS and smaller lateral tibial spine height remain consistently different between ACL-injured and control knees across all pediatric age groups, while MTS differences only emerge in older adolescents [47]. Martin et al. showed that LTS increases in ACL-deficient skeletally immature knees over a mean 9-year follow-up [48]. This raises the hypothesis that chronic ACL deficiency may influence the development of slope morphology, which may be relevant when considering long-term monitoring and secondary injury risk.

### 6.3. Other Anatomical Factors

Multiple additional morphological variables have been proposed, but pediatric evidence remains fragmented. Trochlear morphology, reduced tibiofemoral congruity and many other entities that have been identified as an ACL injury risk factor in the adult literature may represent underappreciated pediatric ACL risk factors [49,50]. Ligamentous laxity, genu recurvatum and an increased Q angle have also been proposed as risk factors [10]. A recent study showed that each one-unit increase in valgus was linked to a greater likelihood of sustaining an ACL injury [51].

### 6.4. Biomechanical and Neuromuscular Factors

Rapid growth phases in children and adolescents are accompanied by transient impairments in postural stability and natural proprioceptive difficulties [52] which lead to decreased neuromuscular control of knee motion, differences in muscle recruitment and timing, and deficits in core- and lower-extremity strength, balance and flexibility [10].

## 7. Management of ACL Tears in Pediatric Patients

**Figure 1:**
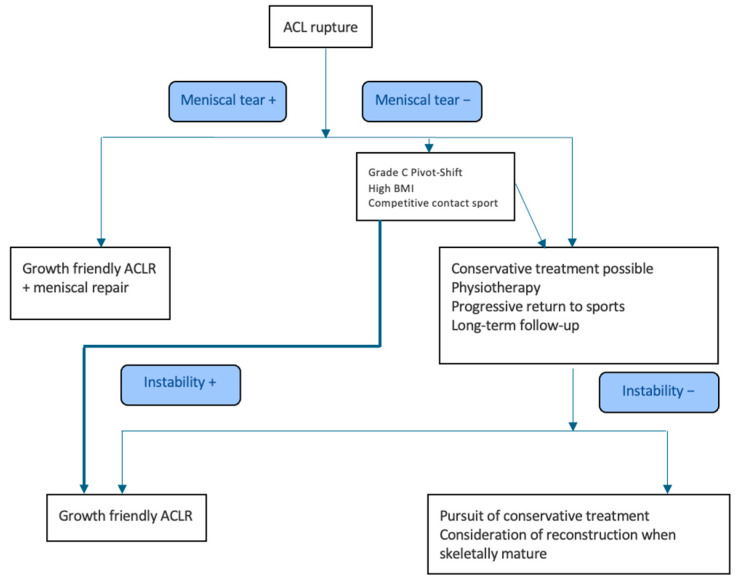
Decisional algorithm after ACL rupture. ACL, anterior cruciate ligament; BMI, body mass index; ACLR, anterior cruciate ligament reconstruction.

In pediatric populations, ACL injuries are especially concerning when joint instability persists over time. This substantially increases the risk of subsequent meniscal and cartilage damage, potentially leading to early-onset osteoarthritis [53,54]. Consequently, when surgical criteria are met, early anterior cruciate ligament reconstruction (ACLR) in children and adolescents has progressively gained acceptance. In the past, surgical treatment was commonly delayed. Orthopedic surgeons preferred non-surgical management (splinting, rehabilitation, and prolonged restriction of sports activities) until skeletal maturity was reached. At that point, ACLR was considered safer. However, this strategy discourages our young patients to participate in sports activities. It also has shown to have a significant psychological impact leading to frustration, isolation, lower self-esteem, depression, etc. [55]. To date, concerns remain about potential growth-related complications in young patients undergoing ACLR. Currently, the International Olympic Committee (IOC) consensus advises surgical management when an ACL rupture is associated with cartilage or meniscal injury, recurrent instability, non-acceptable limitation to practice sports or activities and participation in high-level contact and pivot sports [56]. Conservative management is warranted for all other cases. When managing an ACL rupture in pediatric populations, the initial consideration is thus the presence of associated meniscal injury, necessitating prompt surgical intervention. In the absence of concomitant damage, a nonoperative approach may be pursued, involving structured physical therapy and progressive return-to-sport (RTS). If knee stability is maintained, this strategy can be continued. Conversely, persistent instability despite adequate rehabilitation warrants consideration of surgical treatment. Meniscal injury can be increased up to 60–70% with lasting instability. In the latter case, delayed surgical intervention is associated with gradual increase in the risk of medial meniscal tears [57], primarily in male and obese patients [58] and possibly in patients with varus-aligned knees [59]. Delay > 12 months multiplied the risk of medial meniscal tear requiring surgery by 4.2 [26] and the risk of a bucket-handle tear [60]. According to Grassi et al. patients who underwent surgery within 3 months of injury had fewer meniscal lesions than those treated after more than 6 months. Lesions were less severe compared with patients operated on after 12 months [61]. Moreover, based on expert consensus, early surgical management may be proposed upfront in patients presenting with significant rotational instability (pivot-shift grade C) [62] or elevated body mass index (BMI) (Figure 1). Grassi et al. recently proposed a complementary validated decisional algorithm, the BABY-knee [63]. It is a 6-item-based system (including MRI and patient characteristics) resulting in a 10-point scoring system. Importantly, the overall goal of pediatric ACL injury treatment, whether it is conservative or surgical, is to restore stability while being as safe as possible regarding remaining growth, thereby reducing the risk of further associated lesions and allowing to resume usual daily and sporting activities [23,64,65]. Finally, a crucial aspect of care is thorough discussion with the patient and their parents to clearly outline the available treatment options and ensure that the selected strategy aligns with their goals and expectations.

## 8. Conservative Management of ACL Tears in Pediatric Patients

Conservative management remains a valid treatment option in children and adolescents with complete or partial ACL tears who do not exhibit instability. This approach is particularly appropriate for patients with low functional demands or those participating in non-pivoting or non-contact sports. The commonly recommended protocol begins with acute care aimed at controlling pain and swelling. It is followed by a supervised rehabilitation program lasting at least 12 weeks and often extending up to one year. RTS should not be guided by a fixed timeline, but by the achievement of well-defined functional benchmarks, notably strength and hopping tests showing less than 10% asymmetry [66]. However, pediatric rehabilitation remains poorly described and is mainly extrapolated from the adult literature. This is problematic because growing children and adolescents already face inherent proprioceptive challenges, as the growth spurt is associated with temporary deficits in postural control [52]. When combined with ACL deficiency, these factors may create a fundamentally different neuromuscular dynamic compared with adults. While functional bracing may be recommended upon resuming activity, there is no research regarding its efficacy in children and adolescents with ACL injuries. Furthermore, the sole randomized trial involving adults reported only subjective improvements [67]. Follow-up should be rigorous and structured, with active assessment of persistent instability. Regular MRI follow-up could be considered to detect new meniscal lesions, as recurrent episodes of instability may be subtle. Grassi et al. suggest an annual MRI surveillance [68]. However, the role of routine serial MRI remains an area of limited evidence and heterogeneous practice with no consensus regarding timing nor intervals and no evidence on cost-effectiveness. Resuming cutting and pivoting sports must be approached with great caution, making it essential to recommend a shift toward safer activities. However, this strategy may not be acceptable for competitive patients who are fully committed to returning to their preferred sport. Success rates of non-operative treatment vary according to age. In a prospective cohort of 46 children aged ≤12 years treated according to a structured conservative algorithm, 78% did not require reconstruction at 2 years after their ACL injury [69]. In a retrospective series of 53 patients treated conservatively, 21 patients (40%) ultimately underwent reconstruction and 19 (36%) reported residual instability. The presence of initial clinical instability was found to be predictive of poor tolerance of the conservative strategy [70]. Recent data showcase persisting instability in 20–100% of non-operative cases [64,65]. Evaluation of contralateral injury is not well-documented and described in up to 17% of cases [71].

## 9. ACLR in Pediatric Patients

### 9.1. Evaluation of Skeletal Maturity

Assessment of skeletal maturity is essential when planning ACLR in children and adolescents, as the knee substantially contributes to lower-limb growth [29]. Bone age can be determined using hand or elbow radiographs (Greulich and Pyle [72] or Sauvegrain methods [73] respectively). The Greulich and Pyle method may be considered as outdated and non-universally applicable, as based on radiographs of Caucasian patients in Ohio from 1930 to 1940. The onset of iliac crest ossification (Risser stage 1) signals the nearing end of lower-limb growth [74]. Pubertal markers such as menarche in girls and Tanner stage 4 development in boys further indicate approaching skeletal maturity. Nevertheless, Tanner staging demonstrates a weak association with skeletal maturity [75] and assessments performed by orthopedic surgeons lack sufficient reliability and accuracy [76]. Optimal accuracy is best achieved by combining multiple indicators. However, to reduce healthcare expenditure and avoid unnecessary radiation in pediatric patients, it is essential to evaluate the role of knee MRI and radiographs for determining skeletal maturity. Fabricant et al. recently exposed the strengths and weaknesses of eight knee-radiograph-based and four knee-MRI-based assessment methods of skeletal maturity [77]. Knee MRI offers an excellent inter and intra-observer reliability to evaluate skeletal maturity [78].

### 9.2. Technique and Graft Selection

**Figure 2:**
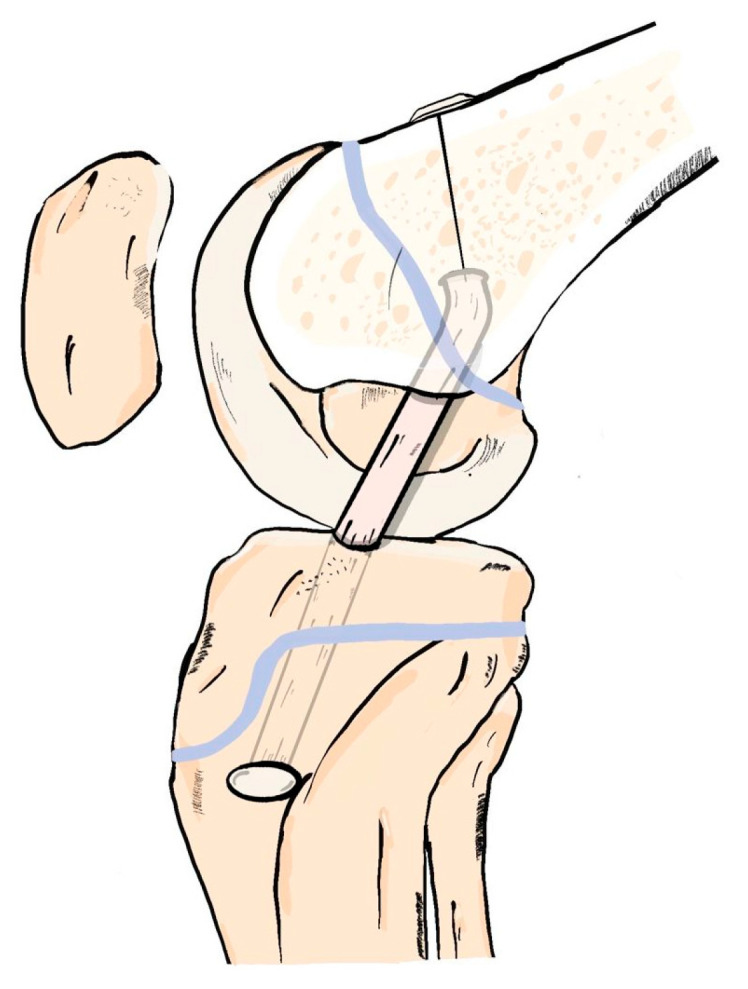
Sagittal illustration of a transphyseal anterior cruciate ligament reconstruction with femoral and tibial tunnel trajectories crossing open physes. Physes are delineated in blue.

Surgical approaches have progressively evolved to minimize the risk of physeal injury and subsequent growth disturbances, while ensuring an anatomic positioning of the graft. Contemporary techniques primarily differ regarding tunnel positioning relative to the physis, graft selection and fixation methods. Irrespective of the chosen approach, it remains essential to consistently address any associated meniscal lesions during arthroscopic intervention. No single pediatric ACLR technique has consistently demonstrated clear superiority over the others for the core endpoints of growth disturbance, graft failure and functional outcomes [29,64,65,79,80]. The available comparative literature is dominated by nonrandomized cohorts in which technique selection is tightly coupled to skeletal maturity [81,82]. Apparent differences between techniques can thus be heavily confounded by baseline skeletal maturity. Other methodological limitations further weaken inference. In a major systematic review focused on growth assessment after pediatric ACLR, only 35% of studies reported follow-up to skeletal maturity and only 12% reported preoperative standing radiographs [81]. As a consequence, low growth disturbance rates may reflect limited surveillance rather than true absence of deformity. Furthermore, the heterogeneity within technique labels can be misleading. The term “physeal-sparing” is often used to group extraphyseal and all-epiphyseal methods. Functional outcomes after pediatric ACLR are generally good and look broadly similar across all techniques [83]. The *transphyseal technique* is widely used nearing skeletal maturity as it involves drilling tunnels through the growth plates and therefore carries a theoretical risk of physeal injury (Figure 2). It allows highly reproducible and anatomic graft positioning but requires strict technical precautions (limited tunnel diameter, atraumatic drilling, avoidance of fixation devices or bone grafts crossing the physis) to minimize physeal injury and bone-bridge formation [84,85,86]. Compared to physeal-sparing techniques, modern series show lower pooled growth disturbances and graft failure rates [81,87], even in younger patients [88]. However, the body of evidence is largely composed of retrospective case series. This challenges the assumption that transphyseal drilling is inherently unsafe and supports a nuanced clinical stance. Transphyseal ACLR may be acceptable even in younger patients when performed meticulously with specific technical adaptations and rigorous postoperative monitoring.

**Figure 3:**
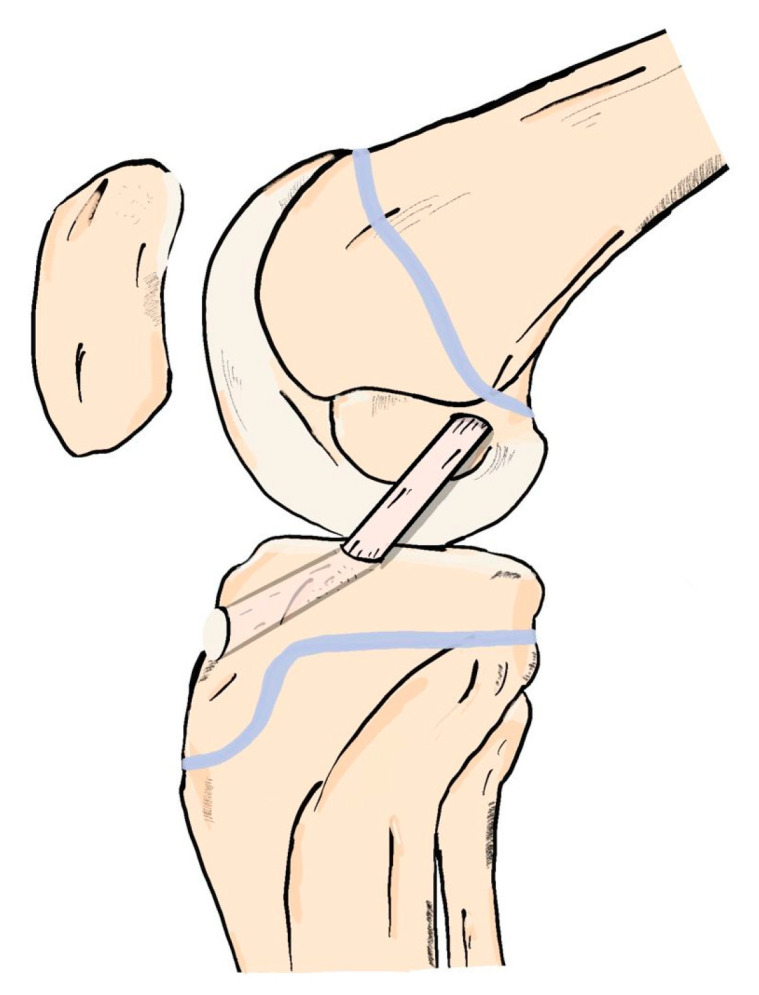
Sagittal illustration of an all-epiphyseal anterior cruciate ligament reconstruction with tibial and femoral epiphyseal entry and pathway. Physes are delineated in blue.

The *all-epiphyseal technique* is typically used in younger children with substantial growth remaining but whose epiphyseal height allows for safe tunnel placement. It involves creating tunnels strictly within the epiphysis, without crossing the growth plates (Figure 3) and requires precise intraoperative monitoring using a fluoroscope [89]. Although conceptually designed to reduce the risk of physeal injury, systematic reviews indicate that growth disturbance rates remain comparable to those of alternative techniques [81,82,90]. This may be attributed to the technical requirements of creating small tunnels within a constrained pathway to approximate accurate anatomical graft placement. Given that the tunnels run tangential to the growth plate, any drilling inaccuracy can lead to unintended physeal involvement across a disproportionately large surface area. Consequently, all-epiphyseal reconstruction should be viewed as a technically demanding option whose success depends heavily on surgical precision and structured growth surveillance, rather than as a guarantee of lower growth disturbance.

**Figure 4:**
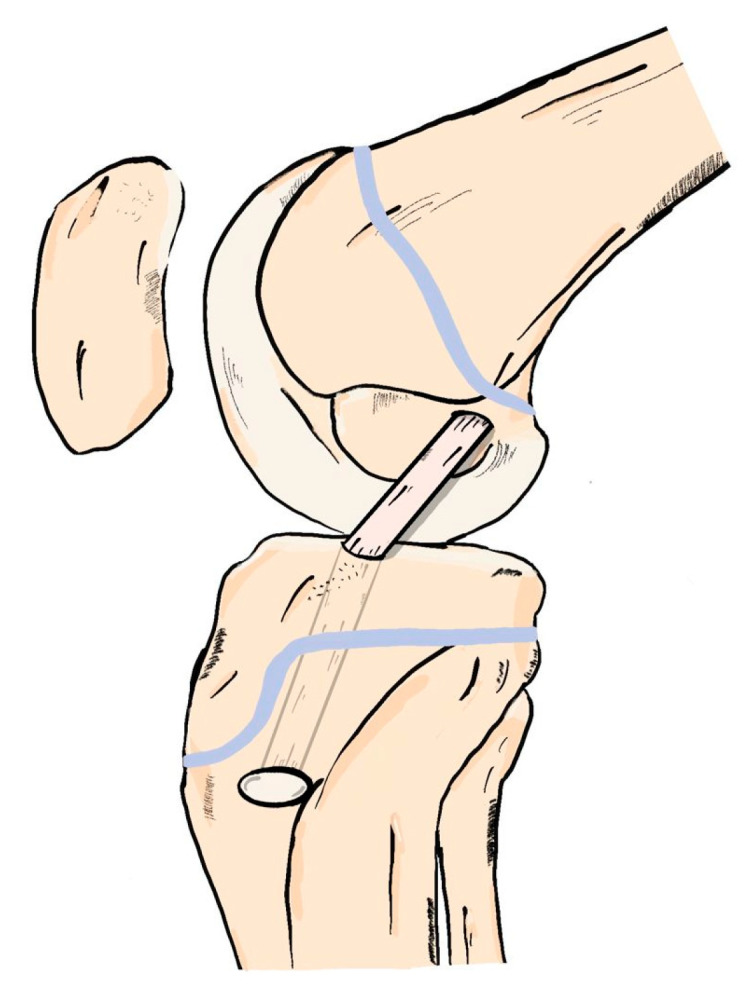
Sagittal illustration of a partial transphyseal anterior cruciate ligament reconstruction with tibial tunnel trajectory crossing the open physis and femoral epiphyseal entry and pathway. Physes are delineated in blue.

A hybrid approach, known as the *partial transphyseal technique*, can also be used [91]. It involves the creation of an epiphyseal tunnel in the femur and a transphyseal tunnel in the tibia (Figure 4). This technique is primarily used at the onset of puberty, when the femoral physis still contributes significantly to limb growth in contrast to the tibial physis. Available comparative syntheses suggest similar outcomes compared to other pediatric ACLR approaches, but its evidence base is particularly vulnerable to selection effects because it targets an intermediate maturity window and is often reported in relatively small cohorts [82]. Partial transphyseal ACLR is a pragmatic compromise for transitional maturity but its comparative safety profile remains uncertain.

**Figure 5:**
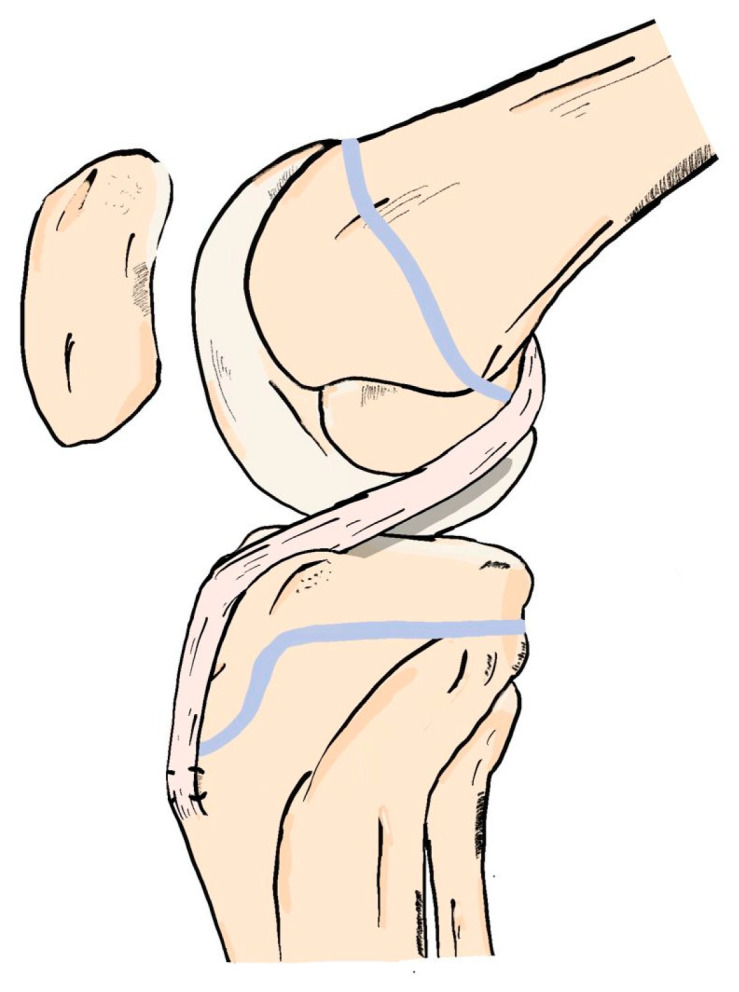
Sagittal illustration of a combined “over the top” (femur) and “over the front” (tibia) anterior cruciate ligament reconstruction. Tunnel-free technique with extra-osseous femoral graft passage and a subperiosteal suture just under the pes anserinus. Physes are delineated in blue.

The fourth option is the so called *“over the top” technique*. The graft is passed behind the lateral condyle and “tied” around it [92]. At the tibial level, an equivalent technique called *“over the front”* allows the graft to pass over the tibial tuberosity, with distal cortical fixation on the metaphysis [93] (Figure 5). These reconstructions are commonly framed as physeal-sparing options for children with substantial growth remaining. They avoid the need for tunnel drilling while providing an intra and extra-articular construct. The main drawback is the difficulty in obtaining perfect anatomical positioning and uniform graft tension. Biomechanical assessments have nevertheless demonstrated a return to almost normal knee kinematics, with broadly comparable stability and functional outcomes with other techniques [94]. Data suggest that growth complications are rare [95], though they still occur occasionally [82]. Extra-physeal techniques are therefore preferred when remaining growth is substantial, but its safety and good functional outcomes have not been proven in maturity-matched comparative studies.

International surveys indicate that ACLR approaches pediatric patients differ considerably depending on skeletal maturity. In North America, prepubescent patients are typically treated with fully epiphyseal tunnels or physeal sparing techniques, whereas transphyseal tunnels are more common in pubescents [7,96]. In Europe, surgeons often use a transphyseal tibial tunnel combined with an epiphyseal femoral tunnel for younger children, moving toward a fully transphyseal approach as adolescents enter puberty [97]. However, there is still no international consensus on which surgical technique is most suitable for specific age groups and the choice of technique largely remains at the discretion of the surgeon. Grassi et al. recently suggested an algorithm based on their own approach [68].

The current literature makes it difficult to provide firm technique-specific recommendations because high-quality comparative studies that evaluate different procedures within truly comparable age and maturity-matched cohorts are lacking. The most defensible conclusion is therefore not that one approach is safer, but that multiple techniques can yield acceptable outcomes when technically well executed and when postoperative growth monitoring is systematic, particularly in younger patients. Future studies should consider randomization of pediatric patients with traumatic ACL ruptures between physeal-sparing and adapted transphyseal techniques [88].

**Figure 6:**
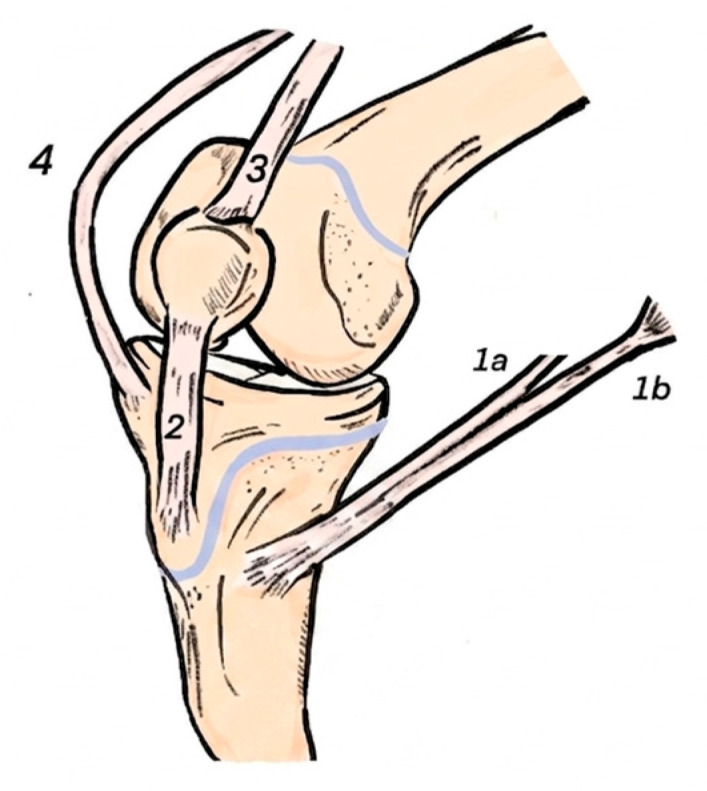
Medial oblique knee illustration of graft options: (1a) gracilis tendon. (1b) Semitendinosus tendon. (2) Patellar tendon. (3) Quadriceps tendon. (4) Iliotibial band. Physes are delineated in blue.

Several types of grafts can be used depending on age and anatomical dimensions [98] (Figure 6). Grafts harvested from the *hamstring tendons* (HTs), whether from the semitendinosus tendon (ST) alone or in combination with the gracilis tendon, are one of the most common choices for pediatric ACLR. This preference is due to the technique’s flexibility and low donor-site morbidity [99]. However, aggregate pediatric data indicate that HT autografts have higher failure rates than other options: 11.8% versus 7.9% for *Bone–Patellar Tendon–Bone* (BPTB) and 2.7% for *quadriceps tendons* (QTs) [100].

The *fascia lata* (FL) or *iliotibial band* (ITB) is used in extra-articular techniques as the modified MacIntosh and Micheli–Kocher techniques [92]. It avoids the need for bone tunnels but may carry donor-site tradeoffs (larger incision and width of the removed fragment leaving an area of insufficient coverage regarding the vastus lateralis muscle, possibly leading to muscle herniation, discomfort and aesthetic concerns [95]). While donor-site morbidity data remain poorly quantified in pediatric populations, evidence in older adolescents and adults indicates that BPTB autografts yield higher local morbidity compared to other alternatives [101]. The patellar tendon (PT) can be utilized in pediatric patients with a modified approach, such as the Clocheville technique [102]. It uses the PT without bone blocks to mitigate the risk of injuring the growth plate. The QT is a promising and increasingly common alternative, with pediatric observational data showing comparable growth disturbances, better functional outcomes and lower graft failure relative to HTs [103]. However, evidence remains scarce compared to other autografts. An available systematic reviews suggests autografts generally provide good outcomes, but none has clear superiority due to a lack of robust comparative studies [99]. *Allografts,* although available, are not the preferred choice in primary pediatric ACLR. Multiple studies report significantly higher rates of re-rupture compared to autografts [104]. Consequently, allografts remain reserved for very specific indications in this patient population. The graft can be fixed using *interference screws*, *cortical fixation* (buttons, staples) or *press-fit* using a bone block [105]. However, in skeletally immature patients, any fixation through a growth plate must be avoided due to the high risk of epiphysiodesis. Therefore, no bone grafts or fixation devices should cross the physis [84].

### 9.3. Clinical and Radiological Follow-Up

Regular surveillance of growth is advised through clinical follow-up combined with standing full-length lower-limb radiographs until skeletal maturity. Although it involves radiation exposure, radiographic monitoring is ideally performed every 6 months during the first 2 postoperative years, followed by annual assessments until skeletal maturity [79]. Early detection of growth disturbances allows prompt and appropriate management. When a clinically meaningful deformity is suspected, further assessment with MRI and/or computed tomography (CT) is warranted to investigate potential growth disturbances [106]. The question of implementing routine annual MRI follow-up to monitor associated lesions remains unresolved, with no evidence or consensus regarding cost-effectiveness and exact timing.

### 9.4. Growth Disturbances After ACLR

Growth disturbances may result from autograft harvesting, tunnel positioning or the method of graft fixation. Chotel et al. described three distinct patterns of growth disturbance [107]. Type A (*Arrest)* corresponds to premature cessation of growth due to focal insult to the physis, resulting in the formation of a physeal bony bridge. Type B (*Boost*) is characterized by accelerated growth, likely driven by increased local vascularity stimulating the remaining open growth plate. Type C (*decelerate)* involves reduced growth velocity attributed to a tenoepiphysiodesis effect or excessive graft tension, ultimately leading to angular deformity. Postoperative growth disturbance is commonly defined as an angular difference exceeding 5° or a mechanical axis deviation of more than 10 mm and/or an LLD greater than 10 mm compared with the contralateral limb [81]. It should be noted that some subclinical growth variations may represent normal physiological differences rather than consequences of the surgical procedure [15]. Reporting and incidence of growth-related complications following ACLR have been evaluated in many studies but remain highly heterogeneous [81,88,108]. Pooled data suggest low reported rates, but also demonstrate that surveillance is frequently insufficient to estimate true incidence [81]. LLD > 10 mm was reported in 2.1% of patients and >20 mm in 0.5%. Among LLDs, shortening is the most common deformity overall, though overgrowth is more frequently reported after all-epiphyseal techniques. Most LLDs involve the femur (83%). The most common angular deformities are femoral valgus (41%), tibial recurvatum (33%) and tibial varus (22%) [81,90]. Overall, the literature consistently indicates that, irrespective of the surgical approach used, postoperative growth disturbances necessitating revision after ACLR are most commonly attributable to technical inaccuracies during the procedure [84,109,110]. However, prospective comparative studies involving large cohorts that examine the association between surgical techniques (moreover age-based surgical techniques) and growth disturbances remain scarce, largely because the selection of the surgical approach is left to the surgeon’s discretion.

### 9.5. Clinical Scores, RTS, Re-Rupture and Contralateral Rupture Rates

All surgical techniques for pediatric ACLR demonstrate good to excellent short- to mid-term clinical outcomes, with high functional scores and RTS rates in early follow-up [80,82,111]. Several systematic reviews showed good and similar functional scores in physeal-sparing, partial transphyseal and complete transphyseal techniques [83,94]. Although functional outcomes mirror those of adult populations [112], graft re-rupture rates remain higher. Indeed, they exceed adult rates irrespective of the surgical technique, representing a continuous challenge for the pediatric population [80,82]. Reported graft re-rupture rates after pediatric ACLR vary by technique, but apparent differences are modest and again likely confounded by skeletal maturity and sport exposure. In a recent systematic review, graft re-rupture incidence was 6% for transphyseal ACLR, 9.8% for partial transphyseal ACLR and 8.1% for physeal-sparing procedures [82]. However, it emphasizes that partial transphyseal and physeal-sparing cohorts were generally younger. An earlier systematic review reported similar re-rupture rates between transphyseal and physeal-sparing approaches but also demonstrated significant demographic imbalance, with physeal-sparing patients being younger [113]. Concomitant *lateral extra-articular tenodesis (LET)* has gained increasing attention as an effective strategy to reduce the risk of graft failure in pediatric patients and allow a quicker, higher-level RTS. Key indications include knee hyperextension greater than 10°; generalized joint laxity; a grade 2 or 3 pivot shift; participation in pivoting, contact, or collision sports; and inadequate neuromuscular control [64]. Systematic reviews and comparative studies confirm that combining LET with ACLR significantly decreases recurrence rates while improving RTS outcomes [114,115]. A recent expert consensus recommended the use of LET in young, physically active patients undergoing ACLR with HT autografts, especially in the presence of pronounced anterior or rotational instability, knee hyperextension, revision procedures or anticipated return to pivoting sports [62]. Rates of contralateral rupture are also significant and rising up to 30% [116]. After ACLR, sex differences may be relevant for contralateral ACL injuries. In a systematic review, ipsilateral graft failure did not differ significantly by sex (9.7% females vs. 7.1% males), whereas contralateral ACL injury was significantly higher in females (22.5% vs. 8.7%), with an odds ratio of 3.0 for contralateral injury in girls [117].

### 9.6. Postoperative Rehabilitation

Postoperative rehabilitation, like conservative treatment, must follow a standardized framework while remaining tailored to each patient. A systematic review by Lorange et al. shows it is still essentially derived from the adult literature. Pediatric RTS criteria are highly variable across studies and optimal criteria remain undefined [118]. The primary goal is to protect the graft during its biological healing phase while restoring range of motion, strength, proprioception, and motor function in order to reduce the risk of re-injury. The integration of a neuromuscular prevention program before and after returning to sport is essential to reduce the risk of recurrence. Returning to pivot/contact and intensive activities is guided strictly by functional criteria. These include a Limb Symmetry Index (LSI) ≥ 90% for muscle strength (quadriceps/hamstrings via isokinetic testing or handheld dynamometer) and jump tests (single leg hop test, triple hop test), satisfactory movement quality, absence of pain and effusion, and adequate psychological readiness [118]. Psychological assessment includes questionnaires on fear of RTS (ACL-RSI) and athletic self-efficacy [119]. The IOC recommends a 9–12-month recovery period before resuming pivot-contact sports. Ideally, athletes should wait 12 months for competitive play [56], as earlier returns are linked to a 3- to 7-fold increase in injury rates [120]. However, these recommendations are primarily based on expert consensus, reflecting limited high-quality evidence. Beyond the initial phase of sport cessation, individualized and long-term rehabilitation strategies tailored to each child’s functional recovery and risk profile may provide a more appropriate approach to minimizing the risk of re-rupture. Finally, pediatric rehabilitation must be multidisciplinary, involving the surgeon, sports physical therapist, sports physician, and, if necessary, a psychologist, while actively including the family in the decision-making process.

## 10. Graft Remodeling After ACLR

### 10.1. General Concepts

After ACLR, the graft undergoes a biological transformation. The tendon progressively remodels to acquire characteristics similar to those of a native ligament. This process is known as “ligamentization” and involves distinct stages: an initial phase of cell necrosis, followed by a phase of proliferation and finally a phase of maturation [121]. This process has been described in animals [122] and humans [123]. In adults, it is relatively well documented and generally lasts 12 months [124], although late remodeling phenomena have been described [125]. Various strategies have been explored to enhance remodeling after ACLR. Biological approaches include the use of growth factors, platelet concentrates such as platelet-rich plasma (PRP) [126], and mesenchymal stem cells [127]. Biomaterials and scaffolds, whether synthetic or biological, are also used to optimize cell colonization and vascularization of the graft [128]. According to a recent systematic review and meta-analysis, adding LET to ACLR does not significantly improve graft maturation [129].

### 10.2. MRI Assessment

**Figure 7:**
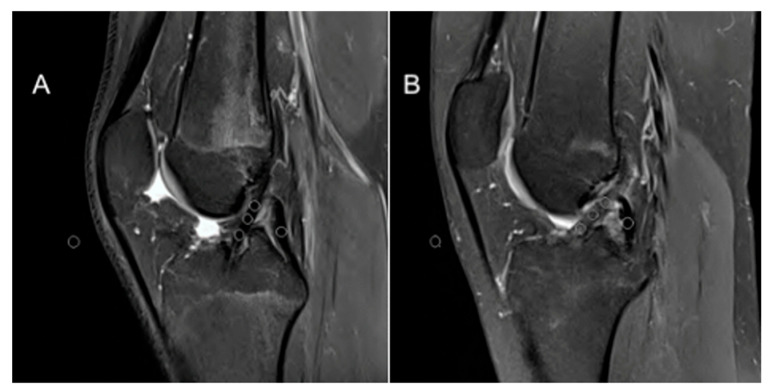
Measurement of SNQ on a sagittal T2 FS MRI. (**A**)—SNQ = 1.2 and Howell grade = I. (**B**)—SNQ = 9.4 and Howell grade = III. SNQ, signal-to-noise quotient; FS, fat saturation; MRI, magnetic resonance imaging. Circles : Placement of regions of interest (ROIs) : 3 ROIs on the intra-articular segment of the anterior cruciate ligament (1 proximal, 1 middle, 1 distal), 1 ROI 2 cm in front of the patellar tendon and 1 ROI on the posterior cruciate ligament.

MRI is a reliable tool and the only non-invasive means to monitor these biological changes. Ligamentization can be measured both quantitatively (using, for example, the *signal-to-noise quotient, SNQ*) and qualitatively (using, for example, *the Howell score*) [130,131] (Figure 7).

In addition, several MRI-based techniques have been developed to assess ACL graft remodeling. *Signal Intensity Ratio (SIR)* compares graft signal to stable reference tissues, typically the PCL or the medial head of the gastrocnemius. A SIR decreasing over time correlates with improved remodeling [132]. MRI sequences including *CISS* [133], UTE-T2* [134], and *Tρ* [135] have also been proposed to evaluate graft structure and composition. *Dynamic contrast-enhanced MRI (DCE-MRI)* evaluates graft revascularization, reflecting the progressive maturation of ligament tissue [136]. *Morphological analysis of the graft*, including measurements of graft CSA or volume, tracks tissue growth and integration over time [136].

### 10.3. Evolution of Graft Maturation

Research indicates that ACL graft ligamentization occurs at a slower pace in pediatric populations compared to adults [137,138,139]. This may be driven by several underlying factors including hormonal influences, inflammatory conditions, vascularization patterns or even specific characteristics of mechanical loads in pediatric patients. There is scarce available literature comparing different surgical techniques in pediatric patients in terms of graft maturation. If RTS timing were to rely primarily on MRI-defined graft maturation, it would often be postponed well beyond typical practice. However, MRI signal of the graft may be variable even in normal adolescent ACLs [140] and no single radiological scoring system is currently reliable enough to dictate RTS independently [141]. Thus, while MRI can support RTS clearance, it should not serve as a stand-alone RTS criterion.

## 11. ACL Repair

The success of contemporary ACL repair is highly dependent on stringent patient selection. While historical attempts in the 1980s yielded poor results due to indiscriminate application, modern surgery utilizes three primary techniques tailored to specific injury types. *Suture-tape augmentation* involves direct suture repair of the torn ACL with high-strength suture tape, particularly indicated for proximal ACL tears (Sherman type I or IIa) with good tissue quality. In adult populations, when applied in these selected cases, suture-tape augmentation has shown good results in terms of residual laxity and patient-reported outcomes [142], but higher failure rates compared ACLR [143], with incidence increasing with younger age [144]. MRI evaluation at 12 months shows repaired ACL tissue demonstrates higher signal intensity compared to reconstruction grafts, indicating ongoing tissue remodeling and healing [145]. Results in pediatric populations remain contradictory. Current research shows a wide variance, with failure rates ranging from 0% in small series [146] to nearly 50% in larger cohorts over a 3-year period [147]. *Bridge-Enhanced ACL Repair (BEAR)* targets midsubstance tears by using a protein-based scaffold and the patient’s own blood to facilitate healing, provided the surgery occurs within 45 days of injury. No data is currently available regarding its use in pediatric populations. *Dynamic Intraligamentary Stabilization (DIS)* utilizes a dynamic polyethylene suture to allow for controlled micromotion during healing and is versatile enough for proximal, midsubstance, or distal tears with adequate tissue. However, this technique is not suitable for pediatric populations because it requires drilling large tunnels for implant placement. To date, ACLR remains the gold standard, particularly in skeletally immature patients [148].

## 12. Prevention of ACL Rupture

In some cases, ACL injuries can lead children and adolescents to definitively quit their sport, or worse, withdraw from sports in general, with potential long-term negative effects extending into adulthood. Furthermore, both conservative and surgical management of ACL injuries come with their own set of challenges and limitations. The issue of sports injury prevention in youth is therefore a societal concern, and consideration must be given to a broader approach putting primary prevention at the center of care for young athletes. Prevention programs such as “FIFA 11+” and their derivatives have shown a substantial reduction in the risk of injuries in pediatric populations [149]. In an international cluster-randomized control trial, implementation of the “11+ Kids” in 7–13 year olds reduced sever injury incidence from 0.33 to 0.15 per 1000 hours, with no occurrence of ACL injuries [150]. Injury mechanisms are not based solely on biomechanical failures, but also on neurocognitive factors (divided attention, visual processing, anticipation, decision-making speed, and sensory integration). Therefore, modern prevention can no longer be limited to correcting alignment or strengthening muscle groups and must incorporate a neuromotor dimension. A systematic review by Watson et al. highlights that neuromuscular training programs significantly reduce the risk of ACL rupture among young athletes [151]. Female children and adolescents should be explicitly highlighted as a higher-risk pediatric subgroup for primary ACL injury and contralateral injury, carrying practical implications for prevention and RTS counselling [152]. Structured neuromuscular training programs substantially reduce ACL injury risk in female/woman/girl athletes, with a FAIR consensus systematic review/meta-analysis reporting a 61% ACL injury reduction [153]. A summary of key prevention programs can be found in Appendix B. Unfortunately, despite demonstrated efficacy under controlled conditions, systematic reviews show that the real-world implementation of prevention programs is hindered by human and contextual factors (lack of time, perceived low utility, variability in prevention programs) [149,154]. It is crucial to act on prevention from an early age, as programs have been shown to be effective in adults as well, but implementation seems even more challenging [155]. Validated strategies to improve adherence to injury prevention programs primarily target organizational and behavioral barriers. Key factors include specific coach training and integrating programs into warm-up routines. Adapting the content to make it more engaging, sport-specific, and motivating, as well as co-constructing programs with end-users (athletes, coaches, clubs), fosters ownership and program sustainability. Using technical feedback (video analysis or real-time correction) enhances execution quality and adherence to the protocol. Finally, considering the sociocultural context and logistical constraints (time, resources, institutional support) is essential to overcome obstacles to implementation. Prevention of ACL rupture in youth patients also requires particular emphasis on limiting early sport specialization and promoting diversified athletic participation [156,157]. Myer et al. highlights that intensive training in a single sport at a young age increases the risk of overuse injuries and impedes optimal neuromuscular development [2]. Instead, encouraging youth athletes to engage in multiple sports fosters broader motor skill acquisition and neuromuscular adaptability, which are protective against ACL rupture [158].

## 13. Conclusions

The management of ACL rupture in the pediatric population remains complex and more constrained by important evidence gaps. While current guidelines offer a valuable foundation for choosing between conservative and surgical treatment, these decision-making frameworks could be further optimized. Crucially, a clear consensus regarding optimal surgical strategies is hindered by the lack of high-quality comparative studies. Rates of graft failure after ACLR and relatively poor outcomes after conservative treatment in young athletes remain notable. This emphasizes the need for continued follow-up into adulthood. Furthermore, rehabilitation is still poorly described in pediatric populations and mainly derived from the adult literature. Population-level risks—such as sex or competition exposure—are well-recognized, but confident individualized prevention requires prospective pediatric data linking modifiable and anatomic factors to incident injuries across maturation stages. Future progress will depend on prospective, multicenter pediatric studies.

## Data Availability

No new data were created or analyzed in this study.

## Abbreviations

The following abbreviations are used in this manuscript:

ACL	Anterior Cruciate Ligament
ACLR	Anterior Cruciate Ligament Reconstruction
BMI	Body-Mass Index
BPTB	Bone-Patellar Tendon-Bone
CSA	Cross-Sectional Area
CT	Computed Tomography
EOS	Electronic Optical Scan
FL	Fascia Lata
FS	Fat-Saturation
HTs	Hamstring Tendons
IOC	Internation Olympic Committee
ITB	Iliotibial Band
LET	Lateral Extra-Articular Tenodesis
LCL	Lateral Collateral Ligament
LLDs	Leg-Length Discrepancies
LSI	Limb Symmetry Index
LTS	Lateral Tibial Slope
MCL	Medial Collateral Ligament
MRI	Magnetic Resonance Imaging
MTS	Medial Tibial Slope
NWI	Notch Width Index
PCL	Posterior Cruciate Ligament
PLC	Postero-Lateral Corner
PRP	Platelet-Rich Plasma
PT	Patellar Tendon
PTS	Posterior Tibial Slope
QT	Quadriceps Tendon
RTS	Return-to-Sport
SIRs	Signal Intensity Ratios
SNQ	Signal-to-Noise Quotient
ST	Semitendinosus Tendon

## Appendix A. Key Clinical Recommendations for ACL Injuries in Pediatric Patients

Initial Assessment & Diagnosis	* Comprehensive medical history and physical examination to identify ACL rupture and associated lesions. * Initial plain radiographs to rule out concomitant fractures, followed by MRI for definitive diagnosis. * Baseline clinical and radiological assessment of lower-limb alignment and length.
Anatomical Risk Factors	* Evaluation of anatomical and neuromechanical risk factors as complementary markers rather than isolated predictive tools.
Skeletal Maturity Assessment	* Adoption of knee radiographs and MRI as preferred modalities for the assessment of skeletal maturity.
Non-Operative Management	* Warranted for patients without functional instability, associated meniscal tears and with preserved functional capacity. * Early transition to surgical stabilization in cases of persistent instability to mitigate the high risk of secondary meniscal damage and early-onset osteoarthritis.
Surgical Management	* Warranted for patients with persisting instability, associated meniscal tears or with unsatisfactory functional capacity. May be considered in patients with significant rotational instability, high BMI or playing in competitive contact sports. * Lack of high-quality comparative studies matching age and maturity cohorts, making technique- or graft-specific recommendations difficult. * However, ACLR remains the gold standard compared to ACL repair.
Follow-up	* Mandatory radiological follow-up to monitor for potential growth disturbances until skeletal maturity. * Mandatory long term rigorous clinical follow-up to account for high graft re-rupture, contralateral rupture and persisting instability rates. * Lack of consensus supporting routine monitoring via follow-up MRI for associated lesions.
Rehabilitation & Return to Sport	* Restriction from recreational sports for at least 9–12 months and competitive sports for a minimum of 12 months post-operatively. * Mandate for strict rehabilitation up to 6–12 months in conservative pathway. * After that, return-to-sport should be guided by functional and psychological benchmarks rather than time-based benchmarks. * Implementation of tailored strategies particularly in higher risk profiles, among other in female patients. * Consideration of MRI as a complementary tool to guide return-to-sport decisions when combined with functional criteria.
	Abbreviations: ACL, anterior cruciate ligament; BMI, body mass index; MRI, magnetic resonance imaging.

## Appendix B. Key Prevention Programs

**Study**	**Population**	**Programs**	**Reported Outcomes**
Watson et al. 2026 [151] (SR/MA)	Athletes <18 years subgroup	– 15–20 minutes warm-up or pre-season training – NMT (sport specific): strengthening + plyometrics + balance/proprioception + flexibility + agility drills + trunk control + landing/cutting technique with feedback (avoid dynamic knee valgus and land jumps with flexed hips and knees)	ACL rupture risk reduction (RR 0.35 [95% CI, 0.22–0.55])
Bullock et al. 2025 [153] (SR/MA)	Female/woman/girl athletes (broad, includes adolescents)	– minimum 10 minutes, twice a week – NMT: balance + strength + agility + change-of-direction with progression from bilateral to single-leg training	Reduction of ACL tear rates by 61% (IRR 0.39 [95%CI 0.25–0.60])
		Abbreviations: SR, systematic review; MA, meta-analysis; NMT, neuromuscular training; RR, risk ratio; CI, confidence interval; IRR, incidence rate ratio.	
